# Therapeutic Modulation of Nitric Oxide Pathways to Address Insulin Resistance in Cardiovascular–Kidney–Metabolic Syndrome

**DOI:** 10.3390/ijms27177701

**Published:** 2026-08-28

**Authors:** Ligia-Maria Ceteraș, Vlad Dumitru Brata, Ioana Dobrotă, Rahela Borbei, Mihai Clim, Teodora-Gabriela Alexescu, Mircea-Vasile Milaciu, Mirela-Georgiana Perne, Cezara-Andreea Gerdanovics, Angela Cozma, Olga-Hilda Orășan

**Affiliations:** 1 Department of Internal Medicine, 4th Medical Discipline, “Iuliu Hațieganu” University of Medicine and Pharmacy, 400015 Cluj-Napoca, Romania; ceterasligia@yahoo.com (L.-M.C.); saracut_ioana_raluca@elearn.umfcluj.ro (I.D.); borbei_rahela@yahoo.ro (R.B.); mihai.clim07@gmail.com (M.C.); teodora.alexescu@umfcluj.ro (T.-G.A.); vasile.milaciu@umfcluj.ro (M.-V.M.); albmirela@yahoo.ro (M.-G.P.); andreea.ceza.irimie@elearn.umfcluj.ro (C.-A.G.); angelacozma@yahoo.com (A.C.); hilda.orasan@umfcluj.ro (O.-H.O.); 2 Faculty of Medicine, “Iuliu Hațieganu” University of Medicine and Pharmacy, 400347 Cluj-Napoca, Romania; 3 Department of Gastroenterology, Regional Institute of Gastroenterology and Hepatology “Prof. Dr. Octavian Fodor”, 400394 Cluj-Napoca, Romania

**Keywords:** cardiovascular–kidney–metabolic syndrome, nitric oxide, insulin resistance, endothelial dysfunction, biomarkers, precision medicine

## Abstract

Cardiovascular–kidney–metabolic (CKM) syndrome encompasses the convergent pathophysiology of obesity, insulin resistance, type 2 diabetes, chronic kidney disease, and cardiovascular disease, conditions whose interactions account for a substantial proportion of cardiovascular morbidity and mortality despite contemporary guideline-directed therapy. Nitric oxide (NO) pathway dysfunction constitutes a unifying mechanism across this continuum, linking endothelial dysfunction, impaired insulin signaling, and multiorgan injury through endothelial NO synthase (eNOS) uncoupling, increased arginase activity, asymmetric dimethylarginine (ADMA) accumulation, and paradoxical inducible NO synthase (iNOS)-driven nitrosative stress. Established cardiometabolic therapies—sodium-glucose cotransporter-2 (SGLT2) inhibitors, glucagon-like peptide-1 receptor agonists (GLP-1 RAs), renin–angiotensin–aldosterone system (RAAS) inhibitors, statins, and metformin—improve NO signaling indirectly through reductions in oxidative stress and inflammation yet fail to fully restore NO bioavailability and leave substantial residual cardiovascular and renal risk unaddressed. Direct NO-restoring strategies, including soluble guanylate cyclase (sGC) modulators, arginase inhibition, ADMA-lowering approaches, and microbiome-targeted interventions, demonstrate mechanistic promise in preclinical and early translational studies but currently lack outcome-level evidence. Biomarkers of NO pathway dysfunction—ADMA, flow-mediated dilation (FMD), the tetrahydrobiopterin–dihydrobiopterin (BH4/BH2) ratio, cyclic guanosine monophosphate (cGMP), and endothelial microparticles (EMPs)—offer a foundation for patient phenotyping but remain insufficiently standardized for clinical use. A NO-centered framework provides a biologically coherent model for understanding residual cardiometabolic risk; its translation into personalized therapy will require validated biomarker panels and biomarker-guided outcome trials.

## 1. Introduction

Cardiovascular–kidney–metabolic (CKM) syndrome reflects the convergence of obesity, insulin resistance, type 2 diabetes mellitus, chronic kidney disease (CKD), and cardiovascular disease (CVD). The global prevalence of obesity has nearly tripled over the past five decades; the number of adults living with diabetes has exceeded 500 million, and CKD affects approximately 10–15% of the adult population. Individuals with coexisting metabolic, renal, and cardiovascular abnormalities have a substantially greater risk of heart failure, atherosclerotic cardiovascular disease, kidney failure, and all-cause mortality than those with a single disease entity [1,2,3].

Recognizing the need for an integrated framework, the American Heart Association introduced CKM syndrome as a systemic disorder characterized by pathological interactions among metabolic risk factors, kidney dysfunction, and cardiovascular disease. Its staging system describes a continuum from stage 0, denoting the absence of CKM risk factors, to stage 4, denoting established cardiovascular disease in the presence of metabolic and/or kidney dysfunction. This framework supports earlier identification of high-risk individuals and emphasizes the shared contributions of obesity, insulin resistance, inflammation, endothelial dysfunction, and neurohormonal activation to progressive cardiovascular and renal injury [3].

Nitric oxide (NO) signaling is one of the molecular pathways connecting metabolic dysfunction with cardiovascular and renal injury. NO regulates vascular homeostasis through endothelium-dependent vasodilation, inhibition of platelet aggregation, modulation of inflammation, maintenance of microvascular perfusion, and regulation of mitochondrial function. It also contributes to metabolic regulation by facilitating insulin-mediated glucose uptake, skeletal muscle perfusion, and insulin signaling. Experimental and clinical evidence links reduced NO bioavailability to endothelial dysfunction, impaired glucose disposal, hypertension, vascular remodeling, glomerular injury, and atherosclerosis. However, the timing and relative contribution of NO dysfunction across the CKM continuum remain incompletely established [4,5,6,7].

Several additional knowledge gaps limit clinical translation. Reliable biomarkers of NO bioavailability are lacking, and the mechanisms of impaired NO signaling vary among CKM populations. Direct NO-restoring interventions have also been evaluated predominantly in mechanistic or early-phase studies rather than in large outcome trials. Consequently, the most appropriate targets within the NO–soluble guanylate cyclase–cyclic guanosine monophosphate (NO–sGC–cGMP) pathway, the patients most likely to benefit, and the long-term clinical effects of NO-centered interventions remain uncertain [2,5,8,9].

Previous reviews have examined NO biology in obesity and insulin resistance, cardiorenal physiology, kidney regulation, endothelial dysfunction, or nitrate–nitrite pathways, generally within individual diseases or specific components of NO signaling [6,7,10,11,12,13]. The present review instead applies an integrated CKM framework to connect impaired NO production and bioavailability with endothelial and microvascular insulin resistance, organ-specific injury, and residual cardiovascular and renal risk. It also distinguishes established cardiometabolic therapies with indirect effects on NO signaling from more direct or experimental NO-targeted strategies according to their level of clinical evidence. Finally, it critically evaluates candidate biomarkers for biological phenotyping and therapeutic stratification while emphasizing their current limitations and need for prospective validation. Table 1 positions the scope and contribution of the present review relative to the previous literature.

## 2. Materials and Methods

This article was designed as a narrative review and was not intended to provide a systematic or exhaustive synthesis of all available evidence. No review protocol was registered. PubMed/MEDLINE and Embase were searched for publications from January 1990 to June 2026. Searches combined terms related to NO signaling and insulin resistance, including “nitric oxide,” “insulin resistance,” “cardiovascular-kidney-metabolic syndrome,” “endothelial dysfunction,” “endothelial nitric oxide synthase,” “eNOS,” “cyclic guanosine monophosphate,” “cGMP,” “asymmetric dimethylarginine,” “ADMA,” “arginase,” and related therapeutic terms. Search terms and combinations were adapted to the syntax of each database. Reference lists of relevant publications were also screened manually.

Studies were considered eligible when they addressed NO signaling in relation to insulin resistance, CKM-related pathophysiology, organ-specific injury, biomarkers, or NO-targeted therapeutic strategies. Human, animal, and mechanistic studies were included. Priority was given to randomized controlled trials, systematic reviews, meta-analyses, and clinically relevant observational and mechanistic studies. Publications that did not address the objectives of the review or provided information already adequately represented by higher-level or more directly relevant evidence were excluded.

No new quantitative synthesis was performed, and findings were reported as presented in the cited publications. No formal risk-of-bias or evidence-certainty instrument was applied. Evidence was interpreted according to study design, relevance to the CKM continuum, directness of NO-pathway assessment, and degree of clinical validation. These methodological features should be considered when interpreting the conclusions of this narrative review.

## 3. NO Biology Relevant to Insulin Sensitivity

Nitric oxide contributes to insulin sensitivity through vasodilation, enhanced microvascular perfusion, and direct modulation of insulin receptor signaling. In endothelial cells, insulin activates the phosphoinositide 3-kinase/protein kinase B (PI3K/Akt) pathway, which stimulates endothelial nitric oxide synthase (eNOS) and increases NO production. The resulting improvement in microvascular perfusion facilitates the delivery of insulin and glucose to skeletal muscle, while NO also supports insulin signaling within insulin-responsive cells [17,18,19].

Reduced NO bioavailability may therefore be both a consequence of insulin resistance and a mechanism that contributes to its progression. The underlying defects are heterogeneous and may include reduced eNOS activity, increased oxidative NO scavenging, and eNOS uncoupling. This distinction has therapeutic implications because strategies that increase NO production may be less effective when accelerated NO degradation or impaired downstream signaling is the predominant abnormality [20,21,22,23].

### 3.1. eNOS-NO-cGMP Signaling

The eNOS–NO–cGMP pathway contributes to insulin sensitivity, particularly through its actions in the vasculature and skeletal muscle [24,25,26,27].

Following insulin stimulation, the PI3K/Akt pathway activates eNOS in endothelial cells and increases NO production. NO then diffuses into adjacent vascular smooth muscle cells, where it activates sGC, which catalyzes the conversion of guanosine triphosphate (GTP) to cGMP. Through cGMP-dependent signaling, NO induces vasodilation and improves skeletal muscle perfusion, thereby facilitating the delivery of insulin and glucose to target tissues [28,29].

NO also modulates insulin signaling through cGMP-independent protein S-nitrosylation [18,30,31]. Relevant targets include protein tyrosine phosphatase 1B (PTP1B) and Src homology region 2 domain-containing phosphatases 1 and 2 (SHP-1 and SHP-2), which negatively regulate insulin receptor signaling. Their S-nitrosylation reduces phosphatase activity and can thereby enhance insulin receptor activation, Akt phosphorylation, and glucose transporter type 4 (GLUT4) translocation in endothelial cells and insulin-responsive tissues, including skeletal muscle, liver, and adipose tissue [17,18,19]. These effects support insulin responsiveness [5], although their relative contribution in human CKM syndrome remains incompletely established.

**Figure 1:**
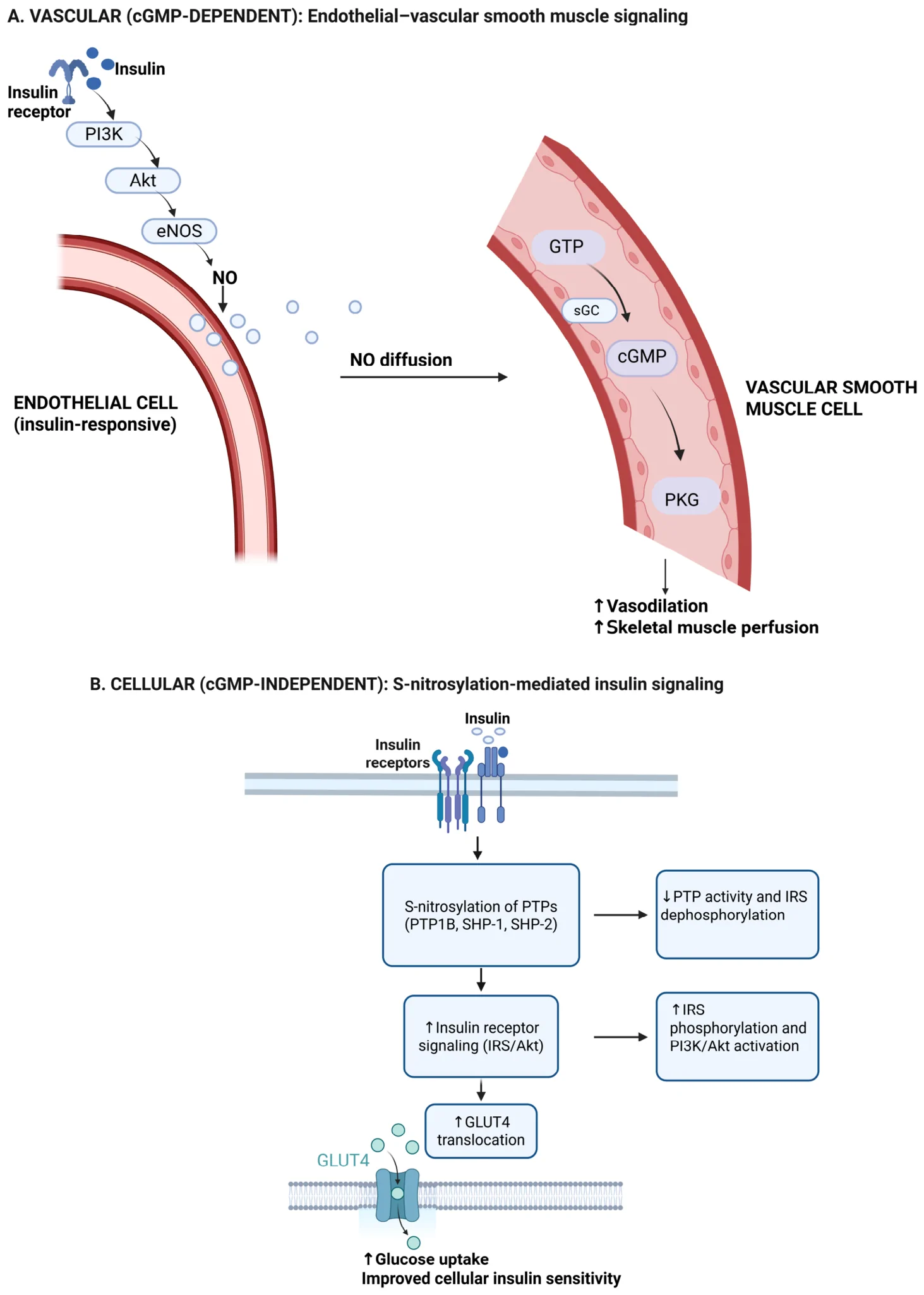
Complementary vascular and cellular mechanisms linking nitric oxide signaling to insulin action. (**A**) Vascular cGMP-dependent mechanism. Activation of endothelial insulin receptors stimulates the PI3K/Akt/eNOS pathway and NO production. NO diffuses into vascular smooth muscle cells, activates sGC, promotes the conversion of GTP to cGMP, and activates PKG, thereby increasing vasodilation and skeletal muscle perfusion. (**B**) Cellular cGMP-independent mechanism. NO-mediated S-nitrosylation of PTP1B, SHP-1, and SHP-2 inhibits protein tyrosine phosphatase activity, limits IRS dephosphorylation, and supports IRS/PI3K/Akt signaling. This promotes GLUT4 translocation, glucose uptake, and cellular insulin sensitivity. Abbreviations: Akt, protein kinase B; cGMP, cyclic guanosine monophosphate; eNOS, endothelial nitric oxide synthase; GLUT4, glucose transporter type 4; GTP, guanosine triphosphate; IRS, insulin receptor substrate; NO, nitric oxide; PI3K, phosphoinositide 3-kinase; PKG, protein kinase G; PTP, protein tyrosine phosphatase; PTP1B, protein tyrosine phosphatase 1B; sGC, soluble guanylate cyclase; SHP-1 and SHP-2, Src homology 2 domain-containing protein tyrosine phosphatases 1 and 2. Created in BioRender. Ceteras, L. (2026) https://BioRender.com/475tk5e.

Thus, NO supports insulin sensitivity through two complementary mechanisms: cGMP-dependent vasodilation and microvascular recruitment, and cGMP-independent modulation of insulin receptor signaling (Figure 1). Dysfunction in either mechanism may impair glucose disposal.

### 3.2. Mechanisms Reducing NO Production and NO Bioavailability in Insulin Resistance

NO bioavailability refers to the amount of biologically active NO available to exert physiological effects and is determined by the balance between its production and inactivation through reactions with superoxide, hemoglobin, and other scavengers [32,33,34]. Unlike NO production, which describes its synthesis by nitric oxide synthases, bioavailability also depends on protection from oxidative degradation and effective signaling in target tissues. It is therefore more informative than production alone when evaluating the relationship between NO and insulin sensitivity [30,31,34].

The mechanisms underlying impaired NO signaling in insulin resistance and CKM syndrome can be broadly divided into those that reduce NO production and those that accelerate its inactivation or disrupt its biological activity.

#### 3.2.1. Mechanisms Reducing NO Production

Under conditions of oxidative stress, cofactor deficiency, or substrate limitation, eNOS can become uncoupled and generate superoxide rather than NO. The relevant determinant is the ratio of reduced tetrahydrobiopterin (BH4) to its oxidized form, dihydrobiopterin (BH2), rather than the absolute BH4 concentration [35,36,37]. Because eNOS binds BH2 and BH4 with comparable affinity, oxidation of BH4 favors the formation of BH2-bound eNOS and superoxide production [36]. Superoxide subsequently reacts with NO to form peroxynitrite, which further oxidizes BH4 and sustains eNOS uncoupling, NO scavenging, and oxidative vascular injury [33,38].

Arginase competes with nitric oxide synthase (NOS) for their common substrate, L-arginine. Arginase 1 (Arg1) is cytosolic and predominantly hepatic, whereas arginase 2 (Arg2) is mitochondrial and is expressed in endothelial cells, vascular smooth muscle, kidney, and skeletal muscle [32]. Increased arginase activity in obesity, insulin resistance, and type 2 diabetes reduces L-arginine availability for NO synthesis [34,36,37]. In insulin-resistant patients with morbid obesity, increased Arg2 expression was associated with impaired endothelium-dependent vasodilation, which improved following L-arginine supplementation or arginase inhibition [39]. Erythrocyte-derived Arg1 may further contribute to systemic L-arginine depletion in type 2 diabetes and heart failure [40].

Asymmetric dimethylarginine (ADMA) accumulates in insulin-resistant states when the activity of dimethylarginine dimethylaminohydrolase (DDAH) is reduced [39,41,42]. Higher ADMA concentrations correlate inversely with insulin sensitivity and endothelium-dependent vasodilation [39,43,44]. ADMA competitively inhibits NOS and may also reduce eNOS phosphorylation through activation of the mitogen-activated protein kinase (MAPK) pathway [45]. DDAH1 accounts for most systemic ADMA clearance, whereas DDAH2 regulates local ADMA concentrations near sites of eNOS activity [46]. By contrast, symmetric dimethylarginine (SDMA) does not directly inhibit NOS and primarily reflects renal clearance rather than NO-pathway dysfunction [47].

#### 3.2.2. Mechanisms Reducing NO Bioavailability

Oxidative stress reduces NO bioavailability through increased production of reactive oxygen species (ROS). Superoxide (O_2_•^−^) rapidly reacts with NO to form peroxynitrite (ONOO^−^) [35,37,38]. Major sources of ROS include nicotinamide adenine dinucleotide phosphate (NADPH) oxidase isoforms 1–5 (NOX1–NOX5), mitochondria during substrate overload, and xanthine oxidase in hyperuricemic states [48,49]. Peroxynitrite further oxidizes BH4, nitrates tyrosine residues, inactivates sGC, and promotes S-glutathionylation of eNOS [5,33,38]. These reactions couple accelerated NO inactivation with progressive eNOS uncoupling and endothelial dysfunction.

Inducible nitric oxide synthase (iNOS; NOS2) represents a distinct mechanism in which NO signaling becomes dysregulated despite increased NO production. It is transcriptionally induced through nuclear factor kappa B (NF-κB)- and Janus kinase/signal transducer and activator of transcription (JAK–STAT)-dependent signaling in response to tumor necrosis factor alpha (TNF-α), interleukin-1 beta (IL-1β), interferon gamma (IFN-γ), and lipopolysaccharide. Unlike eNOS, it generates sustained, high-output NO [50]. In the presence of superoxide, this NO promotes peroxynitrite formation and may induce pathological S-nitrosylation of insulin receptor substrate 1 (IRS-1), Akt inactivation, and kinase nitration, thereby impairing insulin signaling [51]. Nevertheless, iNOS activity is not uniformly pathogenic; in acute settings, iNOS-derived NO contributes to antimicrobial defense and ischemic preconditioning [52].

These mechanisms should not be interpreted as producing a uniform deficiency of NO across all tissues. At the endothelial–vascular interface, eNOS-derived NO may be reduced, whereas inflammatory activation can increase iNOS-derived NO in specific cellular compartments. The biological consequences therefore depend on the cellular source, concentration, spatial distribution, redox environment, and downstream signaling capacity of NO. This distinction may explain why increasing total NO production does not necessarily reproduce the metabolic effects of restoring physiological eNOS-derived signaling.

## 4. NO and Insulin Resistance: Core Mechanisms

In insulin-resistant states, glucotoxicity, lipotoxicity, and chronic low-grade inflammation selectively impair PI3K/Akt/eNOS signaling, which normally couples endothelial insulin action to NO production. The resulting reduction in NO bioavailability becomes functionally important at the endothelial–microvascular interface, where impaired perfusion and substrate delivery contribute to systemic insulin resistance [14,20,29,53,54,55].

### Endothelial and Microvascular Insulin Resistance

Endothelial insulin resistance refers to impaired insulin signaling within vascular endothelial cells, with reduced NO production and endothelial dysfunction as its principal manifestations. Microvascular insulin resistance describes the parallel failure of insulin to recruit capillaries and increase microvascular perfusion in target tissues, particularly skeletal muscle. These processes are closely linked through the PI3K/Akt/eNOS pathway and together influence peripheral glucose disposal [14,15,56].

**Figure 2:**
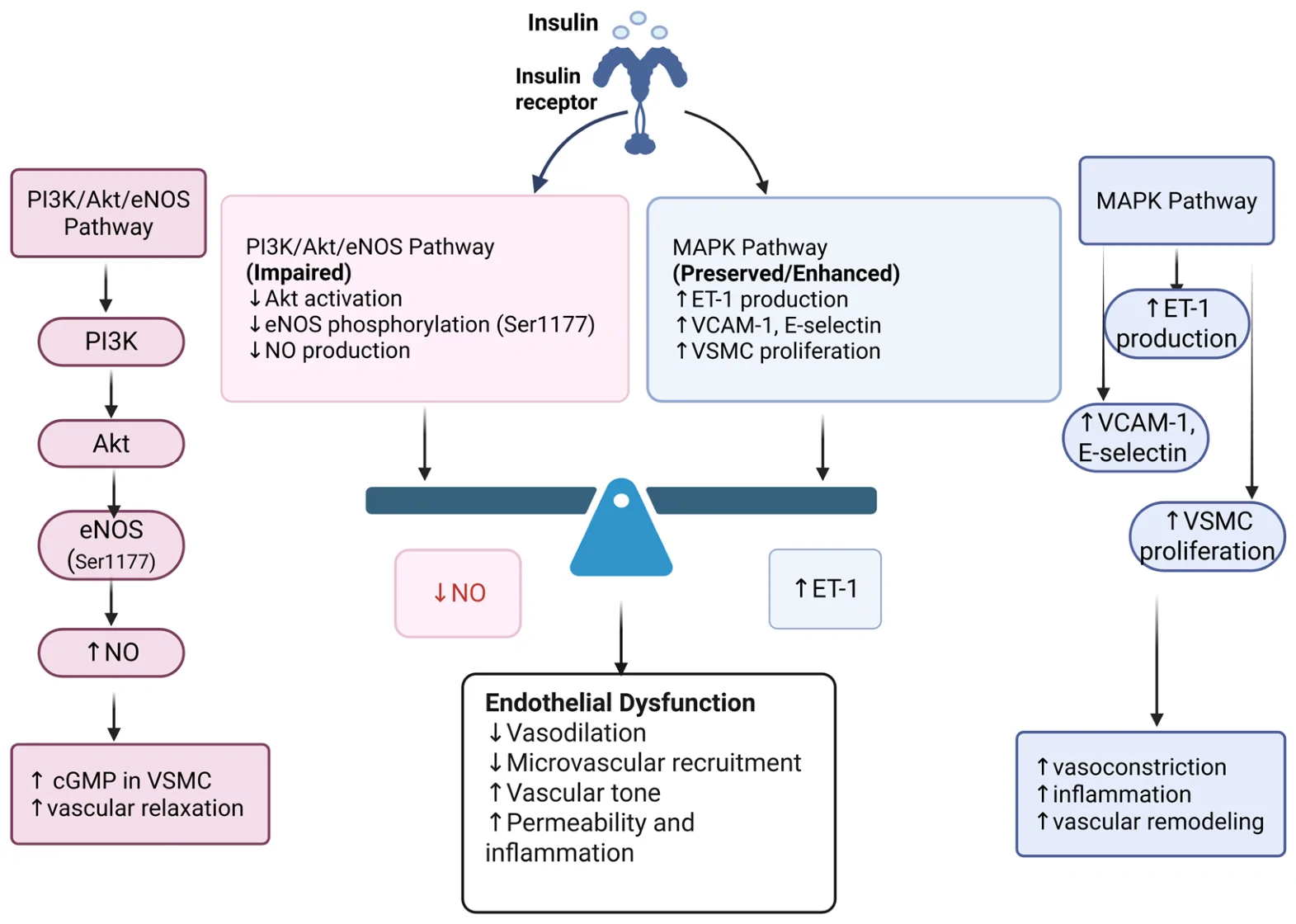
Selective endothelial insulin resistance and imbalance between PI3K/Akt/eNOS and MAPK signaling. Under physiological conditions, insulin activates the endothelial PI3K/Akt/eNOS pathway, promoting eNOS phosphorylation at Ser1177, NO production, cGMP generation in vascular smooth muscle cells, and vascular relaxation. In insulin-resistant states, this pathway is selectively impaired, whereas MAPK signaling remains preserved or becomes enhanced during compensatory hyperinsulinemia. The resulting increases in ET-1 production, VCAM-1 and E-selectin expression, and vascular smooth muscle cell proliferation shift the endothelium toward a vasoconstrictive and pro-inflammatory phenotype. Reduced NO availability combined with increased ET-1 signaling impairs vasodilation and microvascular recruitment while increasing vascular tone, permeability, inflammation, and remodeling. Abbreviations: Akt, protein kinase B; cGMP, cyclic guanosine monophosphate; eNOS, endothelial nitric oxide synthase; ET-1, endothelin-1; MAPK, mitogen-activated protein kinase; NO, nitric oxide; PI3K, phosphoinositide 3-kinase; Ser1177, serine 1177; VCAM-1, vascular cell adhesion molecule 1; VSMC, vascular smooth muscle cell. Created in BioRender. Ceteras, L. (2026) https://BioRender.com/5mbxa3q.

Insulin activates two endothelial signaling branches with different vascular effects. The PI3K/Akt/eNOS branch promotes vasodilation, capillary recruitment, and substrate delivery to skeletal muscle, whereas the MAPK branch promotes endothelin-1 (ET-1) production, vascular smooth muscle proliferation, and expression of adhesion molecules such as vascular cell adhesion molecule 1 (VCAM-1) and E-selectin [29,57]. In insulin resistance, PI3K/Akt/eNOS signaling is selectively suppressed, whereas MAPK signaling remains intact or becomes hyperactive during compensatory hyperinsulinemia [14,29,57]. This imbalance shifts the endothelium from a vasodilatory, anti-inflammatory phenotype toward a vasoconstrictive, pro-inflammatory one, characterized by reduced NO availability, increased ET-1 signaling, elevated vascular tone, and impaired microvascular perfusion (Figure 2).

Physiological concentrations of insulin recruit skeletal muscle microvasculature within 5–10 min, preceding both myocyte signaling and increased glucose disposal. This response is NOS-dependent and can be abolished by the NOS inhibitor N^G^-monomethyl-L-arginine (L-NMMA) [25]. The hemodynamic actions of insulin include relaxation of resistance arterioles, which increases total blood flow; relaxation of terminal arterioles, which recruits previously unperfused capillaries; and expansion of the microvascular surface available for insulin and substrate exchange.

Insulin resistance adds a structural limitation through capillary rarefaction, defined as a sustained reduction in capillary density. This further restricts the surface available for insulin and glucose exchange [58,59]. In mice, muscle-specific deletion of vascular endothelial growth factor (VEGF) reduced skeletal muscle capillary density by approximately 60% and produced insulin resistance independently of primary myocyte signaling defects [58]. These findings indicate that loss of microvascular architecture can contribute to impaired insulin sensitivity.

Endothelial dysfunction and insulin resistance may therefore reinforce each other. Insulin resistance impairs PI3K/Akt/eNOS signaling and reduces NO production, whereas endothelial dysfunction limits microvascular recruitment and substrate delivery, further aggravating peripheral insulin resistance [14]. This bidirectional model suggests that impaired insulin action should not be viewed exclusively as an intracellular defect in insulin-responsive tissues; part of the impairment may arise upstream, at the level of insulin delivery and microvascular recruitment. The endothelial compartment therefore occupies an intermediate position between circulating insulin and its metabolic targets, although it remains unclear how much insulin resistance in humans is attributable to vascular rather than cellular defects.

## 5. Organ-Specific Pathophysiology in CKM Syndrome

The consequences of impaired NO signaling vary across the organs affected by CKM syndrome but share a common pathophysiological pattern: reduced eNOS-derived NO at the endothelial interface, amplification by oxidative and inflammatory stress, and tissue-dependent induction of iNOS in immune or parenchymal cells. The relative contribution of these mechanisms depends on cellular composition, vascular architecture, and metabolic demand, producing distinct patterns of organ injury.

In the vasculature, metabolic risk factors promote oxidative stress, inflammation, eNOS uncoupling, and reduced NO production [10,11,12]. The resulting loss of endothelium-dependent vasodilation increases vascular tone and arterial stiffness and promotes atherogenesis [11,12,13]. With sustained metabolic stress, this functional disturbance progresses toward vascular inflammation, remodeling, and structural atherosclerotic disease [10,11,13].

In the heart, reduced NO bioavailability impairs NO–sGC–cGMP–PKG signaling, promoting cardiomyocyte hypertrophy, myocardial stiffness, diastolic dysfunction, and adverse remodeling [60,61]. Metabolic oxidative and nitrosative stress further promotes inflammation and fibrosis, contributing to the development of heart failure with preserved ejection fraction [60,61,62]. Concurrent impairment of coronary microvascular NO signaling limits the matching of myocardial perfusion to metabolic demand [60,62]. Vascular dysfunction and cardiomyocyte remodeling may consequently reinforce one another within the metabolic HFpEF phenotype.

Renal NO signaling regulates glomerular hemodynamics, tubular transport, and renal blood flow [11,62]. Reduced NO bioavailability favors arteriolar vasoconstriction, impaired natriuresis, sodium retention, and altered glomerular filtration [16,62,63]. Chronic kidney disease further increases ADMA concentrations through impaired renal clearance and altered DDAH activity, thereby aggravating NOS inhibition and endothelial dysfunction [13,16,45,63]. Oxidative stress within the renal vasculature additionally suppresses eNOS activity and shifts the balance toward vasoconstrictor signaling [11,16,63]. These interacting hemodynamic and molecular abnormalities promote glomerular and vascular injury and establish a feedback loop between renal dysfunction and systemic endothelial impairment.

In the liver, inflammatory induction of iNOS generates sustained, high-output NO that promotes S-nitrosylation and tyrosine nitration of insulin-signaling proteins, thereby impairing hepatic insulin action [23,64,65,66]. Excessive iNOS-derived NO can also disrupt autophagy and lysosomal function, contributing to lipid accumulation and metabolic dysfunction [23,66]. By contrast, physiological NO generated by constitutive NOS isoforms supports hepatic glucose and lipid metabolism [23]. Liver-specific iNOS expression is sufficient to induce hepatic insulin resistance in experimental models, supporting a causal role for nitrosative stress rather than a purely associative relationship [64]. Altered S-nitrosoglutathione metabolism may further connect abnormal NO signaling with defective cellular quality control and hepatic steatosis [66].

In skeletal muscle, NO contributes to insulin-stimulated glucose uptake and GLUT4 translocation [67,68,69,70]. Reduced endothelial NO availability impairs microvascular recruitment and capillary perfusion, limiting the delivery of insulin and glucose to myocytes [11,23]. Within muscle cells, iNOS-derived NO and nitrosative stress can directly inhibit insulin signaling [23,68]. Skeletal muscle insulin resistance therefore includes both a vascular defect in substrate delivery and a cellular defect in NO-dependent insulin signaling [67,68,69,70]. Impaired microvascular perfusion may precede and amplify intracellular abnormalities, increasing their contribution to systemic insulin resistance.

In adipose tissue, chronic inflammation and iNOS induction promote nitrosative stress, impaired insulin signaling, lipolysis, free fatty acid release, and systemic inflammation [23,71]. Reduced vascular NO–cGMP signaling may itself promote adipose inflammation, indicating that endothelial dysfunction can actively contribute to adipose tissue pathology [72]. In visceral adipose tissue from individuals with obesity, vascular TNF-α production has been associated with reduced NO availability in small arteries [73]. This interaction is particularly relevant in perivascular adipose tissue surrounding coronary and renal vessels. During obesity, perivascular adipose tissue loses its vasoprotective phenotype and releases pro-inflammatory mediators that impair NO signaling in the adjacent vascular wall, thereby linking adipose dysfunction with coronary and renal microvascular injury [74,75,76].

The principal NO-related mechanisms and their organ-specific consequences across the CKM continuum are summarized in Table 2.

## 6. Clinical Manifestations of Insulin Resistance in CKM Syndrome

The clinical phenotype of CKM syndrome reflects the convergence of insulin resistance and impaired NO signaling across multiple organs. Its manifestations combine metabolic abnormalities, including hyperinsulinemia, dyslipidemia, ectopic adiposity, and hyperglycemia, with vascular abnormalities such as endothelial dysfunction, reduced NO bioavailability, increased vascular tone, and capillary rarefaction. The interaction between these processes contributes to the elevated cardiovascular and renal risk associated with CKM syndrome [14,71].

Atherogenic dyslipidemia is characterized by elevated triglycerides, reduced high-density lipoprotein cholesterol, and increased concentrations of small dense low-density lipoprotein particles. Oxidized low-density lipoprotein further impairs endothelial function by increasing oxidative stress, promoting eNOS uncoupling, and reducing NO bioavailability, thereby accelerating vascular inflammation and atherogenesis [33,38,77,78].

Visceral and ectopic adiposity contribute to insulin resistance through the release of free fatty acids and pro-inflammatory adipokines, including TNF-α, IL-6, and leptin. These mediators increase oxidative stress, impair eNOS activity, and reduce vascular NO bioavailability [65,72,73,79,80,81,82,83]. In the liver, chronic lipotoxicity and inflammation contribute to metabolic dysfunction-associated steatotic liver disease (MASLD) and steatohepatitis (MASH). Increased iNOS activity promotes nitrosative modification of insulin-signaling proteins, whereas reduced sinusoidal NO availability impairs hepatic microvascular function and increases intrahepatic vascular resistance [23,64,65,66,79].

Hypertension in CKM syndrome reflects the interaction of hyperinsulinemia, sympathetic nervous system activation, renin–angiotensin–aldosterone system activity, renal sodium retention, and endothelial dysfunction. Reduced NO bioavailability shifts vascular regulation toward vasoconstrictor signaling mediated by angiotensin II and ET-1, increasing vascular resistance and blood pressure [10,13,14,15,71].

Heart failure with preserved ejection fraction is a prominent cardiac phenotype within the CKM continuum. Systemic and coronary microvascular endothelial dysfunction reduces NO bioavailability and impairs NO–sGC–cGMP–PKG signaling in cardiomyocytes. The resulting titin hypophosphorylation, myocardial stiffness, inflammation, and fibrosis contribute to diastolic dysfunction and adverse cardiac remodeling [60,61,62,84].

NO inhibits platelet activation, adhesion, and aggregation through cGMP-dependent signaling. Impaired NO bioavailability may therefore favor a prothrombotic vascular state in insulin resistance and CKM syndrome [85].

Renal involvement may develop before a measurable decline in glomerular filtration rate. Early abnormalities can include glomerular hyperfiltration, altered tubuloglomerular feedback, activation of the renin–angiotensin system, and progressive endothelial dysfunction. Because the contribution of NO can vary with disease stage and renal compartment, hyperfiltration should not be attributed exclusively to reduced NO availability [10,11,13,16,63]. Albuminuria, quantified using the urinary albumin-to-creatinine ratio (UACR), reflects glomerular and systemic vascular injury and is associated with both cardiovascular and renal risk [86]. UACR is therefore a clinically accessible risk marker that complements, but does not directly measure, NO-pathway dysfunction.

Chronic low-grade inflammation and oxidative stress provide a systemic link among these clinical phenotypes. Elevated C-reactive protein, TNF-α, and IL-6 concentrations and increased NF-κB activity can suppress eNOS expression, promote eNOS uncoupling, and accelerate NO inactivation. Reduced NO bioavailability can, in turn, facilitate endothelial activation, adhesion-molecule expression, and further inflammatory signaling [31,33,34,35,36,37,38,61,82,87,88,89]. This relationship is context-dependent: acute inflammatory responses may be adaptive, whereas persistent metabolic inflammation shifts NO signaling toward oxidative and nitrosative stress. Consequently, suppression of inflammatory signaling alone cannot be assumed to restore endothelial NO bioavailability.

## 7. Therapeutic Strategies Affecting the NO Pathway

Therapeutic interventions relevant to NO signaling range from established cardiometabolic treatments with demonstrated cardiovascular or renal benefits to experimental strategies that directly target NO production, bioavailability, or downstream signaling. The categories below are descriptive and distinguish therapies according to their clinical evidence base and proximity to the NO pathway. They should not be interpreted as a formal evidence-grading system or as proof that NO modulation mediates the observed clinical benefits.

### 7.1. Established Therapies with Cardiovascular or Renal Outcome Evidence

Sodium–glucose cotransporter 2 (SGLT2) inhibitors have demonstrated cardiovascular and renal benefits in trials including EMPA-REG OUTCOME, DAPA-HF, EMPEROR-Preserved, DAPA-CKD, and EMPA-KIDNEY, across populations with type 2 diabetes, heart failure, and chronic kidney disease. Proposed NO-related mechanisms include reduced NADPH oxidase activity, mitochondrial dysfunction, oxidative stress, and inflammatory signaling [90,91].

Glucagon-like peptide-1 receptor agonists (GLP-1 RAs) have demonstrated cardiovascular, metabolic, or renal benefits in LEADER, SUSTAIN-6, REWIND, and FLOW. Mechanistic studies suggest that reduced oxidative stress and inflammation, enhanced eNOS activity, and improved mitochondrial function may contribute to their vascular effects [90,91].

Statins have well-established cardiovascular benefits through lowering of atherogenic lipoproteins and may additionally improve endothelial NO signaling by increasing eNOS expression and activity, limiting eNOS uncoupling, and reducing NADPH oxidase-dependent oxidative stress [92,93,94].

Inhibitors of the renin–angiotensin–aldosterone system reduce cardiovascular and renal risk in appropriately selected patients. Angiotensin-converting enzyme inhibitors, angiotensin receptor blockers, and mineralocorticoid receptor antagonists may preserve NO bioavailability by reducing angiotensin II-mediated oxidative stress, supporting eNOS function, and limiting vascular inflammation [8,95]. Finerenone reduced kidney disease progression and cardiovascular events in patients with chronic kidney disease and type 2 diabetes in FIDELIO-DKD and FIGARO-DKD [96,97].

EMPA-REG OUTCOME, DAPA-HF, EMPEROR-Preserved, DAPA-CKD, EMPA-KIDNEY, LEADER, SUSTAIN-6, REWIND, FLOW, FIDELIO-DKD, and FIGARO-DKD were designed to evaluate clinical outcomes rather than NO-pathway activity. Evidence for NO modulation therefore derives mainly from experimental studies and surrogate measures of endothelial function, oxidative stress, or NO-related signaling [84,90,91,92,93,94,95,96,97]. Direct measurements of NO production or tissue-specific bioavailability were rarely prespecified, so the clinical benefits observed in these trials cannot be attributed specifically to restoration of NO signaling.

Future mechanistic studies should prespecify biochemical or functional measures—such as ADMA, nitrate and nitrite, cGMP, or flow-mediated dilation—and determine whether treatment-induced changes are associated with clinical outcomes.

### 7.2. Clinically Available or Evaluated Interventions with Limited CKM-Specific NO Evidence

sGC acts downstream of NO synthesis and increases cGMP generation through direct stimulation of sGC. Riociguat is indicated for pulmonary arterial hypertension and selected forms of chronic thromboembolic pulmonary hypertension. Vericiguat demonstrated clinical benefit in VICTORIA and is indicated for symptomatic chronic heart failure with an ejection fraction below 45% following a recent worsening event. VICTORIA did not establish that this benefit was mediated by restoration of systemic or tissue-specific NO bioavailability. Neither agent has demonstrated efficacy for insulin resistance or CKM syndrome as an integrated condition, while sGC activators remain investigational [84].

Pioglitazone improves insulin sensitivity and may influence endothelial function through effects on inflammation, adipokine signaling, and eNOS expression. In IRIS, it reduced recurrent stroke or myocardial infarction among patients with insulin resistance after ischemic stroke or transient ischemic attack [98]. This population should not be equated with the broader CKM population, and fluid retention, weight gain, and heart failure risk constrain its clinical use.

Metformin remains widely used for glycemic management in type 2 diabetes. The experimental and mechanistic literature has linked it to AMP-activated protein kinase signaling, enhanced eNOS phosphorylation, reduced mitochondrial oxidative stress, and attenuation of inflammatory pathways [12,85]. However, direct human evidence that restoration of NO signaling mediates its clinical effects is limited, and NO-related biomarkers have not generally been incorporated as prespecified endpoints in metformin trials.

Strategies based on tetrahydrobiopterin include BH4 supplementation, sepiapterin administration, and folate-mediated support of BH4-dependent eNOS coupling. Although these approaches can improve endothelial function or vascular reactivity under selected experimental conditions, clinical evidence remains largely restricted to surrogate outcomes [36,99,100,101]. Their efficacy is also likely to depend on whether BH4 oxidation and eNOS uncoupling are dominant abnormalities in the treated population.

L-arginine and L-citrulline supplementation seek to increase substrate availability for NOS. Some pharmacokinetic studies suggest that L-citrulline can produce more sustained increases in circulating L-arginine because it undergoes less presystemic metabolism [102,103]. Nevertheless, clinical benefits have generally been modest and have focused on blood pressure or endothelial-function endpoints rather than cardiovascular or renal events [13,102,103].

Organic nitrates remain established treatments for symptomatic ischemic heart disease but are not therapies for CKM syndrome itself. Their long-term use may be limited by tolerance, oxidative stress, and nitrate-associated endothelial dysfunction, and they have not demonstrated sustained cardiovascular or renal protection across CKM populations [104,105].

Phosphodiesterase type 5 (PDE5) inhibitors prevent cGMP degradation and thereby amplify signaling downstream of NO. Their effectiveness depends on sufficiently preserved upstream NO–sGC activity, which may be compromised in advanced oxidative vascular disease. Although these agents have established clinical uses outside CKM syndrome, evidence supporting their use for insulin resistance or multiorgan CKM outcomes remains insufficient [106].

### 7.3. Experimental NO-Restoring Strategies

The inorganic nitrate–nitrite–NO pathway provides a NOS-independent route for NO generation through enterosalivary nitrate reduction. Small mechanistic studies have reported improvements in blood pressure, endothelial function, or arterial stiffness, but metabolic effects have been inconsistent and clinical outcome evidence is lacking [10,12,107,108,109]. Chronic nitrate supplementation also failed to improve metabolic health and worsened aspects of disease progression in a mouse model of diet-induced obesity [110]. These findings indicate that the effects of nitrate supplementation depend on biological context, including disease stage, redox environment, diet, and microbiome activity.

Antioxidant interventions such as resveratrol, N-acetylcysteine, and vitamin C have been investigated as indirect means of preserving NO bioavailability and limiting eNOS uncoupling. Although experimental findings are often favorable, clinical studies have produced inconsistent results, possibly because nonspecific suppression of oxidative signaling does not correct every cause of NO-pathway dysfunction [38,111].

Modulation of endogenous S-nitrosothiol metabolism represents another experimental approach. Altered S-nitrosoglutathione reductase activity has been linked to defective autophagy and hepatic insulin resistance in obesity [66]. This finding supports the biological relevance of S-nitrosothiol homeostasis but does not establish the therapeutic efficacy of administering S-nitrosothiols or modifying their metabolism in CKM syndrome.

### 7.4. Biomarkers and Current Limitations of NO Pathway Assessment

Clinical translation of NO-targeted strategies is limited by the difficulty of measuring NO bioavailability in vivo, the heterogeneity of pathway dysfunction across patients, and the absence of validated thresholds for treatment selection or monitoring [84,112]. Because NO is highly reactive and has a short biological half-life, direct measurement is largely restricted to experimental settings. Clinical and translational studies therefore rely on biochemical surrogates and functional vascular measurements, each capturing a different aspect of NO biology [88,113,114].

ADMA reflects endogenous NOS inhibition and has been associated with endothelial dysfunction, insulin resistance, cardiovascular events, and CKD progression [39,41,42,44,45,47]. SDMA does not directly inhibit NOS and primarily provides information about renal clearance and methylarginine metabolism [47]. Plasma nitrate and nitrite, commonly reported together as NOx, provide indirect estimates of systemic NO metabolism but are affected by dietary intake, oral microbiome activity, renal clearance, medication use, and oxidative conditions [10,11,12,88,114]. The BH4/BH2 ratio may reflect eNOS coupling more accurately than either metabolite alone, although its measurement remains largely research-based [35,36,37,38]. cGMP provides information about downstream signaling but is not specific to NO because it can also be generated through natriuretic peptide pathways [60,84,115].

Functional measurements provide complementary information. Flow-mediated dilation assesses endothelium-dependent vasodilatory capacity but is not exclusively determined by NO and is sensitive to vascular remodeling, sympathetic activity, metabolic conditions, operator technique, and pre-test preparation [6,16,116,117]. Pulse wave velocity reflects arterial stiffness and cumulative vascular injury but is not specific to NO dysfunction [16,62]. Circulating endothelial microparticles may indicate endothelial injury or activation, although differences in isolation and quantification methods limit comparability among studies [118,119,120].

Several practical barriers currently prevent routine implementation. Most biochemical markers lack universally accepted reference ranges, standardized sampling conditions, and clinically validated thresholds for diagnosis, risk stratification, or treatment selection. ADMA and SDMA are influenced by kidney function, whereas nitrate and nitrite measurements require control of diet, medication exposure, oral microbiome activity, and renal clearance [10,11,12,39,41,42,45,47,88,114]. Assessment of the BH4/BH2 ratio and endothelial microparticles requires specialized analytical methods and remains insufficiently standardized across laboratories [35,36,37,118,119,120]. Flow-mediated dilation requires strict pre-test conditions, dedicated equipment, technical expertise, and operator training, while its reproducibility is affected by methodological and physiological variability [6,16,116,117]. Cost, assay availability, turnaround time, and the need for repeated standardized measurements create additional obstacles to routine clinical use.

Candidate biomarkers, their potential applications, and their principal limitations are summarized in Table 3.

## 8. Emerging and Future Therapeutic Targets

**Figure 3:**
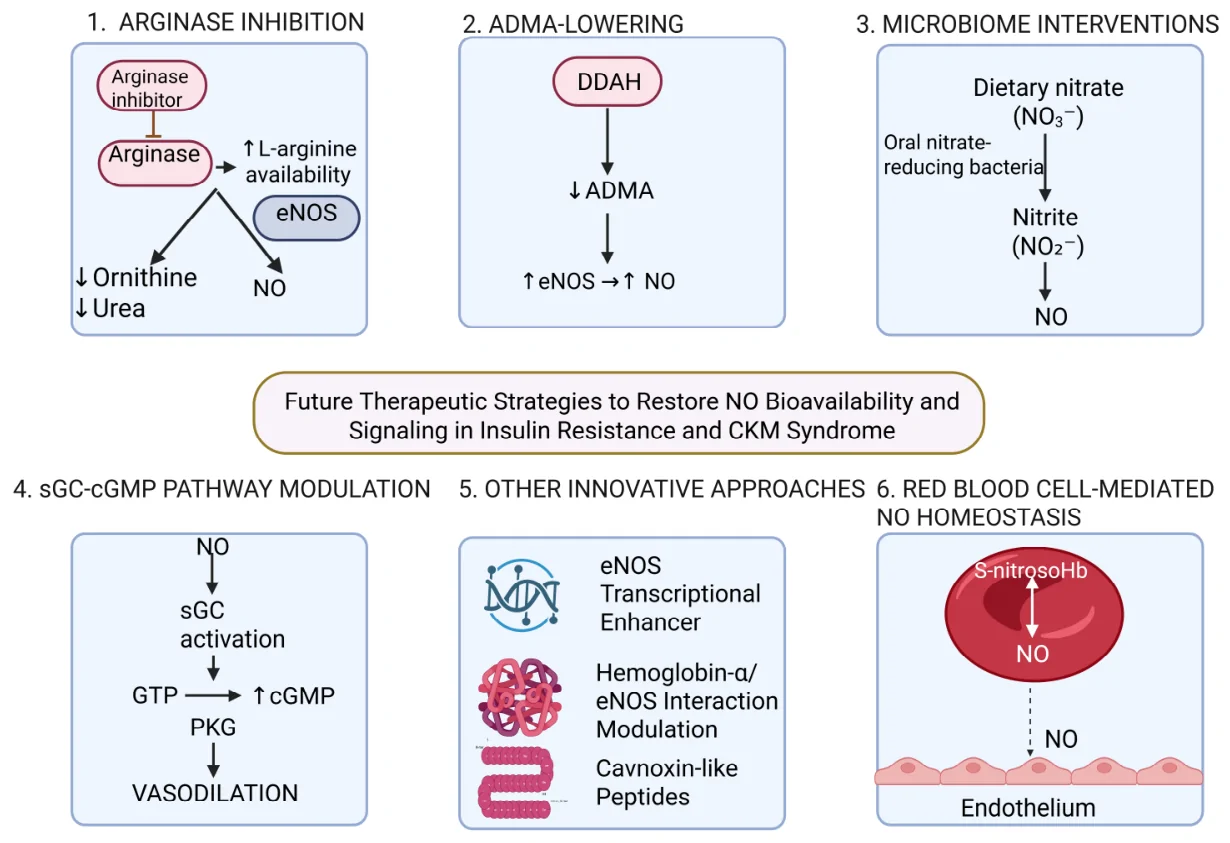
Emerging therapeutic strategies targeting nitric oxide bioavailability and signaling in insulin resistance and cardiovascular–kidney–metabolic syndrome. The proposed approaches include: (1) arginase inhibition to preserve L-arginine availability for eNOS-dependent NO production; (2) enhancement of DDAH-mediated ADMA degradation to relieve endogenous NOS inhibition; (3) microbiome-dependent bioactivation of dietary nitrate through the nitrate–nitrite–NO pathway; (4) pharmacological modulation of sGC–cGMP–PKG signaling to promote vasodilation; (5) enhancement of eNOS expression or activity through transcriptional enhancers, modulation of the hemoglobin-α/eNOS interaction, and Cavnoxin-like peptides; and (6) modulation of erythrocyte-mediated NO homeostasis, including the proposed contribution of S-nitrosohemoglobin to NO bioactivity at the endothelium. These strategies remain predominantly preclinical or at an early translational stage. Abbreviations: ADMA, asymmetric dimethylarginine; cGMP, cyclic guanosine monophosphate; CKM, cardiovascular–kidney–metabolic; DDAH, dimethylarginine dimethylaminohydrolase; eNOS, endothelial nitric oxide synthase; GTP, guanosine triphosphate; Hbα, hemoglobin α; NO, nitric oxide; NO_2_^−^, nitrite; NO_3_^−^, nitrate; PKG, protein kinase G; sGC, soluble guanylate cyclase; SNO-Hb, S-nitrosohemoglobin. Created in BioRender. Ceteras, L. (2026) https://BioRender.com/250lphv.

Emerging NO-directed strategies target substrate competition, endogenous NOS inhibition, microbiome-dependent nitrate reduction, downstream cGMP signaling, and erythrocyte–endothelial interactions. Their translational maturity varies substantially, and most remain supported primarily by preclinical or early mechanistic evidence. The principal emerging strategies are summarized in Figure 3.

Arginase inhibition seeks to preserve L-arginine availability for eNOS. In rodent models of metabolic syndrome and obesity, pharmacological arginase inhibition improved vascular function, reduced blood pressure, and enhanced insulin sensitivity [121,122]. Whether these effects translate into cardiovascular or renal benefits in patients with CKM syndrome remains unknown.

Enhancement of DDAH activity represents a complementary strategy for lowering ADMA. A long-acting recombinant DDAH preparation reduced ADMA, improved endothelial function, lowered blood pressure, and limited ischemia–reperfusion injury in preclinical models [123]. These findings demonstrate target engagement but have not yet been validated clinically.

Pharmacological eNOS transcriptional enhancers, including AVE3085 and AVE9488, increase eNOS expression and may limit eNOS uncoupling in experimental models [78]. Cavnoxin-like peptides are designed to disrupt the inhibitory interaction between caveolin-1 and eNOS, thereby increasing eNOS-derived NO, but evidence remains preclinical [115]. Modulation of the hemoglobin-α/eNOS interaction at myoendothelial junctions offers another potential means of increasing local NO bioavailability, although this approach remains mechanistic [85].

Microbiome-targeted strategies are based on the essential contribution of oral and gut bacteria to bioactivation of dietary nitrate through the nitrate–nitrite–NO pathway [124,125]. Human observational data have associated the nitrite-generating and nitrite-depleting capacity of the oral microbiome with cardiometabolic risk profiles [126]. However, these associations do not establish that probiotics, prebiotics, or other microbiome interventions can restore NO signaling or improve clinical outcomes.

The reduced, heme-containing and oxidized, heme-deficient forms of sGC represent pharmacologically distinct targets for sGC stimulators and activators, respectively [127]. Next-generation agents, including the sGC stimulator praliciguat and the sGC activator ataciguat, are being investigated for broader cardiovascular and metabolic applications, but their efficacy in insulin resistance or CKM syndrome has not been established [115].

Erythrocyte-mediated regulation of vascular NO provides another emerging target. Erythrocytes from patients with type 2 diabetes have been shown to induce endothelial dysfunction through increased arginase I activity in experimental vascular assays [128]. These findings implicate erythrocyte arginase and NO metabolism in vascular dysfunction but do not yet establish a clinically actionable therapeutic strategy [85,128].

## 9. Clinical Implications and Key Messages

NO-pathway dysfunction provides an integrative mechanistic framework for understanding the vascular, metabolic, cardiac, and renal manifestations of CKM syndrome. However, it should not be interpreted as a uniform deficiency state or as a clinically validated basis for treatment selection. Reduced eNOS-derived NO, eNOS uncoupling, ADMA accumulation, increased arginase activity, oxidative NO inactivation, impaired sGC–cGMP signaling, and iNOS-mediated nitrosative stress represent biologically distinct abnormalities that may require different interventions [33,38,39,42,45,64,65,66,84].

Established CKM therapies, including SGLT2 inhibitors, GLP-1 receptor agonists, renin–angiotensin–aldosterone system inhibitors, finerenone, and statins, provide cardiovascular or renal benefits in appropriately selected patients. Experimental and surrogate-endpoint studies suggest that several of these treatments may also improve NO-related signaling, but the major outcome trials were not designed to establish NO modulation as the mediator of clinical benefit [90,91,92,93,94,95,96,97]. Treatment decisions should therefore remain based on demonstrated clinical indications rather than presumed effects on the NO pathway.

Mechanism-based selection of NO-targeted therapy remains a research objective. Strategies that increase NO production may be ineffective when oxidative inactivation or impaired downstream responsiveness predominates, whereas cGMP-directed interventions may bypass some upstream defects. Conversely, excessive iNOS-derived NO requires control of nitrosative stress rather than indiscriminate enhancement of NO production. These distinctions support biological phenotyping but do not yet justify a clinical treatment algorithm.

Available biomarkers also remain insufficient for routine therapeutic selection. ADMA, nitrate and nitrite, the BH4/BH2 ratio, cGMP, flow-mediated dilation, and pulse wave velocity assess different aspects of NO biology, but none directly quantifies tissue-specific NO bioavailability or has validated thresholds for selecting or monitoring therapy [10,11,12,39,45,47,113,114,116,117]. Multimarker panels should therefore be regarded as investigational until their analytical validity, reproducibility, prognostic value, and association with treatment response have been established prospectively.

Future evaluation of NO-based combination therapy should follow a stepwise design. Initial randomized phase II studies should test an NO-targeted intervention as add-on therapy to optimized guideline-directed CKM treatment, using candidate biomarkers for enrichment or stratification while treating them as investigational. Primary objectives should include safety and prespecified target engagement, supported by functional vascular measurements; UACR and other organ-level measures may serve as secondary outcomes but should not be interpreted as direct measures of NO activity. Combination regimens should proceed only after their individual components demonstrate target engagement and acceptable safety and should preferably address complementary defects, such as upstream eNOS dysfunction and impaired downstream cGMP signaling. If early studies demonstrate biological activity, adequately powered trials should evaluate cardiovascular death, heart-failure events, and kidney disease progression. Until such evidence becomes available, NO-targeted and biomarker-guided strategies remain investigational.

## 10. Conclusions

NO-pathway dysfunction provides an integrative framework linking insulin resistance with vascular and multiorgan injury across the CKM continuum. Reduced eNOS-derived NO, eNOS uncoupling, increased arginase activity, ADMA-mediated NOS inhibition, oxidative NO inactivation, iNOS-derived nitrosative stress, and impaired sGC–cGMP signaling may each contribute. Their relative importance varies among organs, disease stages, and metabolic phenotypes, and current human evidence does not establish NO dysfunction as the sole or dominant cause of residual cardiovascular and renal risk.

Contemporary CKM therapies improve clinical outcomes, but these benefits cannot be attributed specifically to restoration of NO signaling. Direct NO-targeted interventions remain predominantly preclinical or at an early translational stage. Further progress requires mechanism-appropriate interventions, standardized and prospectively validated measures of pathway activity, and trials that distinguish molecular target engagement from clinical benefit. NO homeostasis should therefore be viewed as a useful framework for integrating vascular, metabolic, cardiac, and renal pathophysiology and generating testable therapeutic strategies, rather than as an established biomarker-defined treatment paradigm.

## Figures and Tables

**Table 1:** Positioning of the current review relative to the previous literature.

Previous Review	Main Focus	Key Limitations	What the Current Review Adds	References
Sansbury and Hill, 2014	Role of NO in obesity and insulin resistance	Focused primarily on metabolic regulation; predates the CKM syndrome concept and contemporary cardiometabolic therapies	Integrates insulin resistance into the broader CKM continuum and links NO dysfunction to cardiovascular and renal outcomes	[7]
Rajapakse et al., 2015	L-arginine–NO pathway in cardiorenal syndrome	Restricted to heart–kidney interactions; does not incorporate obesity, diabetes, or CKM staging	Expands the framework to include the full cardiovascular–kidney–metabolic spectrum and residual cardiorenal risk	[13]
Carlström, 2021	NO signaling in kidney regulation and cardiometabolic health	Broad review of NO biology with emphasis on renal physiology; not centered on insulin resistance or CKM syndrome	Provides a disease-oriented model linking NO deficiency, insulin resistance, endothelial dysfunction, and CKM progression	[11]
Reviews of nitrate–nitrite–NO biology and NO restoration strategies	Focus on alternative NO-generating pathways and NO replacement approaches	Typically examine isolated NO pathways rather than integrated CKM pathophysiology	Places emerging NO-restoring therapies within a clinically relevant CKM framework and stratifies them according to level of clinical evidence	[10,12]
Reviews of endothelial dysfunction in diabetes and cardiovascular disease	Focus on vascular biology and endothelial impairment	Usually address individual diseases rather than multisystem interactions	Links endothelial dysfunction with insulin resistance, CKD progression, HFpEF, and residual cardiorenal risk	[6,14,15,16]
Current Review	NO pathway dysfunction across CKM syndrome	Narrative synthesis; the proposed biomarker-guided framework requires prospective validation	Integrates molecular mechanisms, organ-specific effects, biomarkers, and NO-targeted therapies according to the available level of evidence	—

Abbreviations: CKM, cardiovascular–kidney–metabolic; NO, nitric oxide.

**Table 2:** Organ-specific mechanisms and consequences of impaired nitric oxide signaling in cardiovascular–kidney–metabolic syndrome.

Organ or Tissue	Principal NO-Related Mechanisms	Main Pathophysiological Consequences	References
Vasculature	Reduced eNOS-derived NO, eNOS uncoupling, and oxidative stress	Impaired endothelium-dependent vasodilation, increased vascular tone and arterial stiffness, vascular remodeling, and atherogenesis	[10,11,12,13]
Heart	Impaired NO–sGC–cGMP–PKG signaling and increased oxidative and nitrosative stress	Cardiomyocyte hypertrophy, myocardial stiffness, fibrosis, diastolic dysfunction, and HFpEF	[60,61,62]
Kidney	Reduced NO bioavailability, ADMA accumulation, altered DDAH activity, and impaired renal hemodynamics	Arteriolar vasoconstriction, sodium retention, glomerular and endothelial injury, and CKD progression	[11,13,16,45,62,63]
Liver	iNOS-derived nitrosative stress, S-nitrosylation and nitration of insulin-signaling proteins, and impaired autophagy	Hepatic insulin resistance, defective cellular quality control, and steatosis	[23,64,65,66]
Skeletal muscle	Impaired microvascular NO signaling and iNOS-mediated disruption of myocyte insulin signaling	Reduced microvascular recruitment, impaired glucose uptake, and peripheral insulin resistance	[11,23,67,68,69,70]
Adipose tissue	iNOS-mediated nitrosative stress, reduced vascular NO–cGMP signaling, and pro-inflammatory signaling from perivascular adipose tissue	Impaired adipocyte insulin signaling, increased lipolysis and inflammation, and coronary and renal microvascular dysfunction	[23,71,72,73,74,75,76]

Abbreviations: ADMA, asymmetric dimethylarginine; CKD, chronic kidney disease; cGMP, cyclic guanosine monophosphate; DDAH, dimethylarginine dimethylaminohydrolase; eNOS, endothelial nitric oxide synthase; HFpEF, heart failure with preserved ejection fraction; iNOS, inducible nitric oxide synthase; NO, nitric oxide; PKG, protein kinase G; sGC, soluble guanylate cyclase.

**Table 3:** Candidate biochemical and functional markers of nitric oxide-pathway dysfunction in cardiovascular–kidney–metabolic syndrome.

Biomarker	Pathway Assessed	Potential Application	Main Limitations	References
ADMA	Endogenous NOS inhibition	Candidate marker of endothelial dysfunction and cardiovascular and renal risk	Influenced by kidney function and analytical method; no validated threshold for treatment selection	[39,41,42,44,45,47]
SDMA	Methylarginine metabolism and renal clearance	May support interpretation of methylarginine concentrations in CKD	Does not directly inhibit NOS; strongly influenced by kidney function	[47]
NOx	Systemic NO metabolism	Pharmacodynamic assessment in mechanistic and interventional studies	Influenced by diet, oral microbiome activity, kidney function, medication exposure, and pre-analytical conditions; does not distinguish NO sources	[10,11,12,88,114]
BH4/BH2 ratio	Redox balance and eNOS coupling	Mechanism-specific assessment of eNOS uncoupling	Requires specialized assays and careful sample handling; lacks standardized clinical thresholds	[35,36,37,38]
cGMP	Downstream cyclic-nucleotide signaling	Potential pharmacodynamic marker of sGC-targeted interventions	Not specific to NO because natriuretic peptides also stimulate cGMP production; does not identify tissue-specific signaling	[60,84,115]
EMPs	Endothelial injury and activation	Complementary indicator of endothelial dysfunction	Isolation, phenotyping, and quantification methods are insufficiently standardized; not specific to NO dysfunction	[118,119,120]
FMD	Endothelium-dependent vasodilatory capacity	Functional assessment of vascular phenotype and treatment-associated changes	Not determined exclusively by NO; requires strict pre-test conditions, technical expertise, and operator-dependent measurement	[6,16,116,117]
PWV	Arterial stiffness and cumulative vascular injury	Functional assessment of vascular damage and cardiovascular risk	Not specific to NO dysfunction; influenced by age, blood pressure, and structural vascular remodeling	[16,62]

Abbreviations: ADMA, asymmetric dimethylarginine; BH2, 7,8-dihydrobiopterin; BH4, tetrahydrobiopterin; cGMP, cyclic guanosine monophosphate; CKD, chronic kidney disease; EMPs, endothelial microparticles; eNOS, endothelial nitric oxide synthase; FMD, flow-mediated dilation; NO, nitric oxide; NOS, nitric oxide synthase; NOx, nitrate/nitrite metabolites; PWV, pulse wave velocity; SDMA, symmetric dimethylarginine; sGC, soluble guanylate cyclase.

## Data Availability

No new data were created or analyzed in this study. Data sharing is not applicable to this article.

## Abbreviations

The following abbreviations are used in this manuscript:

ADMA	asymmetric dimethylarginine
Akt	protein kinase B
Arg1	arginase 1
Arg2	arginase 2
BH2	dihydrobiopterin
BH4	tetrahydrobiopterin
cGMP	cyclic guanosine monophosphate
CKD	chronic kidney disease
CKM	cardiovascular–kidney–metabolic
CVD	cardiovascular disease
DDAH	dimethylarginine dimethylaminohydrolase
DDAH1	dimethylarginine dimethylaminohydrolase 1
DDAH2	dimethylarginine dimethylaminohydrolase 2
EMPs	endothelial microparticles
eNOS	endothelial nitric oxide synthase
ET-1	endothelin-1
FMD	flow-mediated dilation
GLP-1 RA	glucagon-like peptide-1 receptor agonist
GLUT4	glucose transporter type 4
GTP	guanosine triphosphate
HFpEF	heart failure with preserved ejection fraction
IFN-γ	interferon gamma
IL-1β	interleukin-1 beta
IL-6	interleukin-6
iNOS	inducible nitric oxide synthase
IRS	insulin receptor substrate
IRS-1	insulin receptor substrate 1
JAK/STAT	Janus kinase/signal transducer and activator of transcription
L-NMMA	N^G^-monomethyl-L-arginine
MAPK	mitogen-activated protein kinase
MASH	metabolic dysfunction-associated steatohepatitis
MASLD	metabolic dysfunction-associated steatotic liver disease
NADPH	nicotinamide adenine dinucleotide phosphate
NF-κB	nuclear factor kappa B
NO	nitric oxide
NO_2_^−^	nitrite
NO_3_^−^	nitrate
NOS	nitric oxide synthase
NOS2	nitric oxide synthase 2, the gene encoding iNOS
NOx	nitrate and nitrite metabolites
NOX1–NOX5	NADPH oxidase isoforms 1–5
O_2_•^−^	superoxide anion
ONOO^−^	peroxynitrite
PDE5	phosphodiesterase type 5
PI3K	phosphoinositide 3-kinase
PKG	protein kinase G
PTP	protein tyrosine phosphatase
PTP1B	protein tyrosine phosphatase 1B
PWV	pulse wave velocity
ROS	reactive oxygen species
SDMA	symmetric dimethylarginine
Ser1177	serine residue at position 1177
sGC	soluble guanylate cyclase
SGLT2	sodium–glucose cotransporter 2
SHP-1	Src homology region 2 domain-containing phosphatase 1
SHP-2	Src homology region 2 domain-containing phosphatase 2
SNO-Hb	S-nitrosohemoglobin
TNF-α	tumor necrosis factor alpha
UACR	urinary albumin-to-creatinine ratio
VCAM-1	vascular cell adhesion molecule 1
VEGF	vascular endothelial growth factor
VSMC	vascular smooth muscle cell
